# Integrated Microbiome and Metabolomics Analysis Reveals That *Ganoderma lucidum* Triterpenoids Ameliorate Colitis Associated with the Modulation of the Gut Microbiota and Metabolic Profiles

**DOI:** 10.3390/foods15112016

**Published:** 2026-06-04

**Authors:** Weiling Guo, Ye Li, Jinzhi Han, Xueyan Liu, Li Ni

**Affiliations:** 1 Institute of Food Science and Technology, College of Biological Science and Engineering, Fuzhou University, Fuzhou 350108, China; weilingguo2021@163.com (W.G.); hjz419@fzu.edu.cn (J.H.); 2 College of Chemical Engineering, Fuzhou University, Fuzhou 350108, China; 3 Fujian Xianzhilou Biological Science and Technology Co., Ltd., Fuzhou 350108, China; lee@xianzhilou.com; 4 School of Pharmacy, Fujian Medical University, Fuzhou 350112, China

**Keywords:** *Ganoderma lucidum*, triterpenoids, intestinal barrier, gut microbiota, metabolomics

## Abstract

Colitis is a global health challenge that severely impairs quality of life, necessitating effective dietary interventions. This study investigated the protective effects of *Ganoderma lucidum* triterpenoids (GLTs) on pathological symptoms, inflammatory responses, and gut microbiota dysbiosis in a dextran sulfate sodium (DSS)-induced murine model. Our results demonstrated that GLT intervention significantly attenuated the disease activity index (DAI), prevented colon shortening, and fortified gut barrier integrity through upregulating the transcription of tight junction proteins. GLT inhibited the secretion of pro-inflammatory cytokines (TNF-α, IL-1β, and IL-6) and bolstered antioxidant defenses (CAT) by controlling the *TLR4*/*NF-κB* pathway and upregulating the *Nrf2* pathway. Furthermore, 16S rRNA sequencing and non-targeted metabolomics revealed that GLT reshaped the gut microbial landscape (enriching *Enterorhabdus* and *Lachnospiraceae NK4A136 group*) and reconfigured amino acid metabolism to restore colonic homeostasis. Collectively, these findings highlight the potential of GLT as a functional food ingredient to prevent colitis, potentially linked to the modulation of the microbiota–metabolite–immune interplay, offering a novel nutritional strategy for inflammatory bowel disease management.

## 1. Introduction

Ulcerative colitis (UC) is a prominent form of inflammatory bowel disease, which is characterized by abdominal pain, unintended weight loss, and bloody diarrhea [1]. Over recent decades, the prevalence of UC has plateaued in many developed countries, whereas a marked increase in incidence has been observed in numerous developing countries [2]. The etiology of UC is multifactorial, including the convergence of genetic predisposition, environmental triggers, and intestinal microbial imbalance. Although the pathogenesis of UC is complex, inflammation and oxidative stress are always considered primary pathogenic factors in the onset of colitis. Sustained oxidative stress and inflammatory responses destroy the integrity of the epithelial barrier and increase the risk of colorectal cancer [3]. Furthermore, damage to the intestinal epithelial barrier causes pathogen penetration and inflammatory cell infiltration, which further promote the accumulation of reactive oxygen species [4]. Some drugs with antioxidant and anti-inflammatory effects are extensively used to improve colitis in clinical practice, such as corticosteroids, 5-aminosalicylic acid, glucocorticoid and sulfasalazine. However, these drug treatments are restricted because of their adverse reactions, including drug tolerance, diarrhea, bellyache, and gut microbiota disorder [5]. Therefore, there is an urgent need to identify safer and more effective therapeutic agents with minimal side effects to suppress inflammation and oxidative stress in colitis.

*Ganoderma lucidum* (also known as Rui Cao in ancient China, and Youngzhi in Korean) is a famous edible fungus in China, which has been widely applied as functional food and traditional medicine. *G. lucidum* is rich in various active ingredients, such as polysaccharides, triterpenoids, protein sterol, and alkaloids [6]. Among these, triterpenoids are a widely dispersed class of secondary metabolites from *G. lucidum*. They exhibit a series of beneficial influences on host health because of their unique antioxidant and anti-inflammatory properties. Triterpenoids isolated from *G. lucidum* (GLT) are primarily highly oxygenated lanostane derivatives, generally exhibiting high polarity with low volatility. GLT intervention improved age-related cognitive injury by elevating the abundance of Eubacterium lentum and consequently promoting the product of serine [7]. Our previous study found that GLT (consisting of ganoderic acid A, ganoderic acid C6, ganoderic acid G, and ganoderic acid Me) effectively enhanced the antioxidant enzyme activity and regulated the gut microbiota composition in hyperlipidemic rats [8]. Nevertheless, whether GLT can improve colitis through regulating the gut microbiota and inflammatory response remains to be further investigated.

This study aimed to investigate the efficacy of GLT on the gut barrier and gut microbiota in DSS-induced colitis mice. We focused on how GLT regulates inflammation, oxidative stress, tight junction proteins, intestinal microbiota, and metabolism. However, it remains unclear whether GLT protects against colitis through these coordinated mechanisms. We hypothesized that GLT attenuates colitis by restoring tight junction proteins, suppressing the *TLR4*/*NF-κB* pathway, upregulating the *Nrf2* pathway, and reshaping the gut microbiota and its metabolites. The results of the present study not only elucidate the benefits of GLT in rehabilitating gut homeostasis imbalance but also provide a theoretical foundation for using GLT as a functional food ingredient to ameliorate colitis.

## 2. Materials and Methods

### 2.1. Preparation of GLT

*G. lucidum* was purchased from Xianzhilou Biological Science and Technology Co., Ltd. (Fuzhou, China) and dried using an oven (Jinghong Experimental Equipment Co., Ltd., Shanghai, China). Dry *G. lucidum* was smashed and then it was filtered using an 80-mesh sieve (Dongyuan Machinery Co., Ltd., Xinxiang, China). GLT was obtained according to our previous report [8]. The sample was extracted thrice with 95% ethanol (*v*/*v*), each time soaked for 6 h. Ethanol extraction solution was combined, and then the supernatant was harvested using centrifugation (11,000× *g*, 12 min, 1 °C). The ethanol in sample was evaporated under reduced pressure using a rotary evaporator (Dalong Xingchuang Instrument Company, Beijing, China) at 60 °C. The resulting concentrate was then transferred to an evaporating dish and heated in an 80 °C water bath to remove residual solvent, ultimately yielding the alcoholic extract of *G. lucidum*. The crude extract was redissolved in 60% ethanol, and the triterpenoid fraction was purified using AB-8 macroporous adsorption resin. After achieving adsorption equilibrium, the target compounds were eluted with 90% ethanol. The resulting eluate was further refined using a chloroform/methanol solution to eliminate impurities. Finally, the solvents were removed via rotary evaporation and an 80 °C water bath, followed by repeated recrystallization to obtain the purified *G. lucidum* triterpenoids (GLTs).

### 2.2. Animals and Experimental Design

Twenty-four male C57BL/6J mice were obtained from Wushi Laboratory Animal Co., Ltd. (Fuzhou, China), and maintained under standard environmental conditions (22 ± 1 °C, 53 ± 3% humidity, and a 12 h light/dark cycle). All procedures were approved by the Animal Ethics Committee of Institute of Food Science and Technology (No. FZU-IFST-2025-108). All mice acclimated for 7 days prior to experiments, and were randomized into three groups (*n* = 8): the normal control group (NC) with oral gavage of 0.2 mL of sterile water, the model group (DSS) with oral gavage of 0.2 mL of sterile water, and the experimental group (GLT) with oral gavage of 0.2 mL of GLT solution (150 mg/kg/day). The experimental dosage was determined based on the recommended daily intake for adults (15 g/60 kg body weight) specified in the Chinese Pharmacopoeia, and also took into account the extraction yield of GLT (9.8 ± 0.18%). Apart from the NC group, mice in all groups were freely supplied with 3% DSS for days 7 to 14 to induce colitis (Figure S1). All mice were euthanized after fasting for 10 h at the end of the experiment, and the organs were harvested and stored at −80 °C for subsequent analysis.

### 2.3. Disease Activity Index (DAI)

From days 8 to 14 of the experiment, the DAI score (including weight loss rate, fecal state, and hematochezia status) of each mouse was measured. Colon tissue was harvested after the mice were sacrificed, its length was measured using a ruler, and images of colons were recorded using a digital camera.

### 2.4. Oxidative Stress and Inflammatory Cytokine Assay

Fresh colon tissue was transferred to a clean centrifuge tube and homogenized in RIPA lysis buffer using a high-throughput tissue homogenizer (Xinzhi Biotechnology Co., Ltd., Ningbo, China). After centrifugation (15,000× *g*, 12 min, 2 °C), the supernatant was harvested. The concentration of antioxidant parameters (T-AOC, SOD, GSH, and CAT), oxidative and inflammatory markers (MPO and MDA), pro-inflammatory cytokines (including TNF-α, IL-1β, and IL-6), and anti-inflammatory cytokine interleukin-10 [IL-10] was detected by commercial assay kits (Enzyme-linked Biotechnology Co., Ltd., Shanghai, China).

### 2.5. Histopathology

The colon tissue was excised, and then it was rinsed with phosphate-buffered saline (PBS) and fixed in 4% formaldehyde, followed by dehydration, permeabilization, paraffin embedding, sectioning, and staining with hematoxylin and eosin (H&E), alcian blue (AB), and periodic acid-Schiff (PAS).

### 2.6. Gene Expression Analysis

Total mRNA of colon tissue was extracted by the Trizol reagent (Takala, Dalian, China), and its purity and concentration were detected using NanoDrop and Agilent 2100 Bioanalyzer (Thermo Fisher Scientific Inc., Waltham, MA, USA). mRNA reverse transcription into cDNA was carried out using the PrimeScript™ RT kit (Takala, Dalian, China). qPCR reactions were performed by CFX96 Touch real-time PCR detection (Bio-Rad, Waltham, MA, USA) based on the SYBR Green Premix Pro Taq qPCR kit (TaKaRa, Tokyo, Japan), and the primer sequences are provided in Table S1.

### 2.7. Short-Chain Fatty Acid Analysis

Short-chain fatty acid (SCFA) levels (including acetic, propionic, butyric, isobutyric, valeric, and isovaleric acids) were measured by gas chromatography based on our previously described method [9]. Briefly, cecal content samples were freeze-dried, weighed, and soaked in saturated NaCl solution (0.5 mL). After standing for 30 min, the samples were homogenized using a high-throughput tissue homogenizer (Xinzhi Biotechnology Co., Ltd., Ningbo, China). Next, 10 μL of 10% (*v*/*v*) sulfuric acid solution was added with the samples, followed by thorough vortexing. After centrifugation (15,000× *g*, 12 min, 2 °C), the supernatant was harvested and extracted with anhydrous ether (0.8 mL). Following vigorous shaking, the upper organic phase was transferred to a clean EP tube containing 0.2 g of anhydrous sodium sulfate. After a brief final vortex to ensure dehydration, the supernatant was harvested and transferred to autosampler vials. Finaly, the cecal SCFA concentrations were measured using gas chromatography.

### 2.8. Fecal Microbiota Composition Analysis

Total genomic DNA was extracted by a kit (TIANamp Stool DNA Kit, TianGen Biotech, Chongqing, China). The sample was subsequently subjected to PCR amplification using barcoded primers (338F and 806R). Different fragments in the PCR product were separated using 1.3% agarose gel, dissociated, and recycled using the PicoGreen dsDNA assay kit (Merck KGaA, Darmstadt, Germany). The concentration of each sample was detected using a microplate reader (BioTek, Thermo Fisher Scientific Inc., Waltham, MA, USA), and then all samples were mixed based on the equal amounts of each sample. Sequencing libraries were used on the illumina novaseq 6000 platform (Mingke Biotechnology Co., Ltd., Hangzhou, China).

Raw sequencing data were processed using the Xshell platform based on Quantitative Insights into Microbial Ecology 2, and low-quality sequences were removed for subsequent analysis. After chimera removal, the contigs were then clustered into operational taxonomic units (OTUs) and OTU abundance was estimated, which was used for further analysis. Principal coordinates analysis (PCoA) based on the Bray–Curtis distance and PERMANOVA that was conducted was used to analyze the whole-gut microbiota composition, and statistical analyses (such as *t*-tests) were applied to screen taxa with significant differential abundance between the different groups. In addition, the correlation between genus-level biomarkers and colitis-associated phenotypic parameters was evaluated and visualized using Spearman’s correlation analysis and TBtools software (v 2.0). The raw 16S rRNA sequencing data were deposited in the National Genomics Data Center (PRJCA065187).

### 2.9. Colon Metabolomics Analysis

Colon contents were cooled using a freeze-dryer (Boyikang Instrument Co., Ltd., Changsha, China), and weighed using an analytical balance (Mettler Toledo International Limited, Zurich, Switzerland), and then 0.9 mL of extraction solution was added (acetonitrile:water = 4:1). After vortexing for 90 s, the supernatant was harvested by centrifugation (13,000× *g*, 12 min, 2 °C), and then freeze-dried by a vacuum freeze centrifuge. Samples were dissolved using extraction solution, and then the supernatant was harvested by centrifugation (13,000× *g*, 15 min, 0 °C). A quality control (QC) sample consisted of 10 μL of every sample, which was used to monitor the stability of instruments. The colon metabolomic profile was separated and detected using UPLC Q Exactive-MS/MS equipped with an ACQUITY UPLC HSS T3 column (2.1 × 100 mm, 1.8 μm). The mobile phases consisted of 0.1% formic acid in water (A) and 0.1% formic acid in ACN (B). The raw data were aligned using Compound Discoverer software (version 3.8) based on chromatographic peak detection, alignment, and normalization; mzCloud search; mzVault search; mass list match; and chemSpider search. Normalized data were further analyzed by MetaboAnalyst 6.0 (https://www.metaboanalyst.ca/, 5 February 2026). The raw metabolomics data were deposited in the National Genomics Data Center (OMIX017199).

### 2.10. Statistical Analysis

All data are presented as mean ± SEM, and statistical analyses were carried out using GraphPad Prism software (version 9.0) based on Student’s *t*-test, while analyses requiring comparisons of more than two groups utilized one- or two-way Analysis of Variance (ANOVA) followed by Tukey’s post hoc test when necessary.

## 3. Results

### 3.1. GLT Alleviated the Clinical Symptoms of Colitis Mice

**Figure 1:**
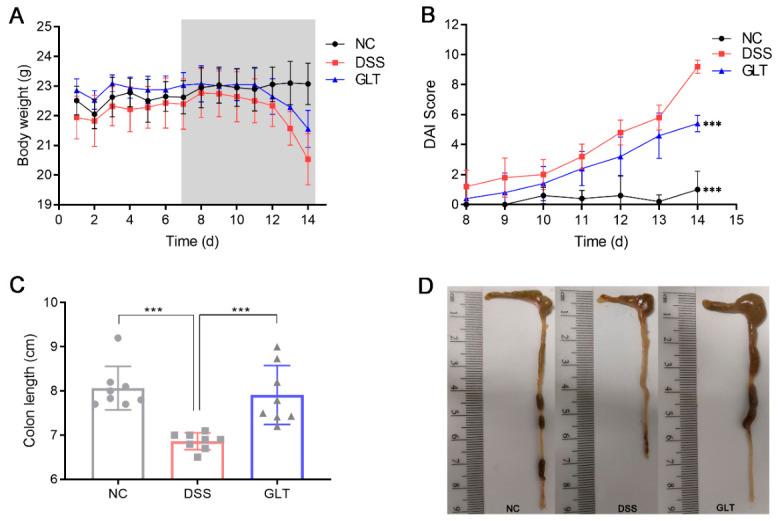
Effects of GLT on clinical phenotypes in DSS-treated colitis mice. (**A**) Dynamic changes in body weight, *n* = 8; (**B**) dynamic changes in DAI scores, *n* = 8; (**C**) colon length, *n* = 8; (**D**) representative photographs of colonic segments. *** *p* < 0.001 represents significance between each group compared with the DSS group.

DSS is widely employed to induce experimental colitis models, as it effectively mimics the pathological features characteristic of human colitis. In the present study, DSS was applied to establish the model and explore the improvement effects of GLT. As shown in Figure 1A, the body weight of mice in the NC group slightly increased, whereas the body weight of mice in the DSS group continuously reduced. Interestingly, the oral administration of GLT significantly inhibited the DSS-caused body weight loss. The DAI score is extensively used in both clinical practice and animal experiments to assess the severity of colitis. As depicted in Figure 1B, DSS-treated mice exhibited a high DAI score compared to healthy mice (*p* < 0.001). However, GLT treatment effectively mitigated this increase (*p* < 0.001). Furthermore, DSS treatment disrupts colonic architecture and exacerbates colon atrophy. As presented in Figure 1C,D, DSS-induced colitis mice exhibited markedly shortened colons (*p* < 0.001), but GLT intervention dramatically prevented the shortened colons (*p* < 0.001). These data indicate that GLT exerts a protective effect against colonic injury in colitis mice.

### 3.2. GLT Alleviated the Oxidative Stress and Inflammation in Colitis Mice

**Figure 2:**
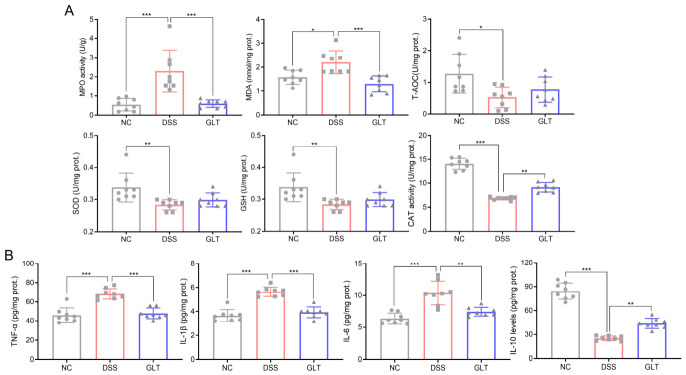
Regulatory effects of GLT on colonic redox balance and inflammatory responses. (**A**) Evaluation of colonic oxidative stress status, *n* = 8; (**B**) profiles of colonic inflammatory cytokines, *n* = 8. * *p* < 0.05, ** *p* < 0.01, *** *p* < 0.001 represent significance between each group compared with the DSS group.

Excessive oxidative stress and an imbalance of pro- and anti-inflammatory mediators are driving factors for colitis pathogenesis. Therefore, the antioxidant and anti-inflammatory effects of GLT were explored in colitis mice. As illustrated in Figure 2A, DSS treatment significantly triggered colonic oxidative stress, characterized by a marked elevation in MPO and MDA concentrations (*p* < 0.05) and a concurrent depletion of antioxidant defense components, including T-AOC, SOD, GSH, and CAT (*p* < 0.05). Notably, GLT intervention effectively mitigated the accumulation of these oxidative markers and partially restored CAT activity (*p* < 0.01), although no significant differences were observed in colonic SOD, T-AOC, and GSH levels between the DSS and GLT groups (*p* > 0.05). Additionally, the levels of pro-inflammatory cytokines (TNF-α, IL-1β, and IL-6) were dramatically elevated, while the anti-inflammatory cytokine IL-10 was significantly reduced in the DSS group compared to the NC group (*p* < 0.001, Figure 2B). Conversely, GLT treatment effectively reversed these trends by significantly reducing pro-inflammatory cytokine concentrations and enhancing IL-10 production (*p* < 0.01). Collectively, these data suggest that GLT exerts a protective effect against colitis by attenuating inflammatory responses and oxidative stress.

**Figure 3:**
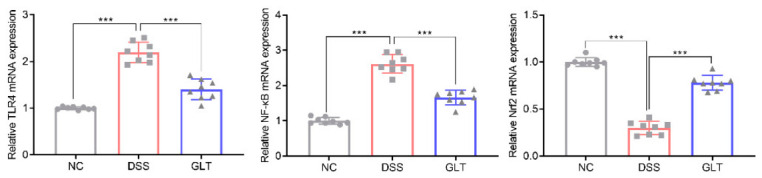
GLT suppressed TLR4/NF-κB activation and upregulated Nrf2-mediated antioxidant signaling in colitis mice, *n* = 8. *** *p* < 0.001 represents significance between each group compared with the DSS group.

To investigate whether GLT intervention modulates inflammatory responses and oxidative stress via the *TLR4*/*NF-κB* and *Nrf2* pathways, the mRNA expression levels of key colonic genes were quantified using RT-qPCR (Figure 3). Compared with the NC group, DSS administration led to a significant upregulation of *TLR4* and *NF-κB* transcription, alongside a marked downregulation of *Nrf2* transcription (*p* < 0.001). Conversely, GLT intervention significantly attenuated the transcriptional levels of *TLR4* and *NF-κB* while enhancing *Nrf2* transcription in colitis mice (*p* < 0.001). These results suggest that GLT ameliorates DSS-induced colonic inflammation and oxidative stress by inhibiting the *TLR4*/*NF-κB* signaling pathway and activating the *Nrf2* defense system.

### 3.3. GLT Improved the Gut Barrier Injury in Colitis Mice

**Figure 4:**
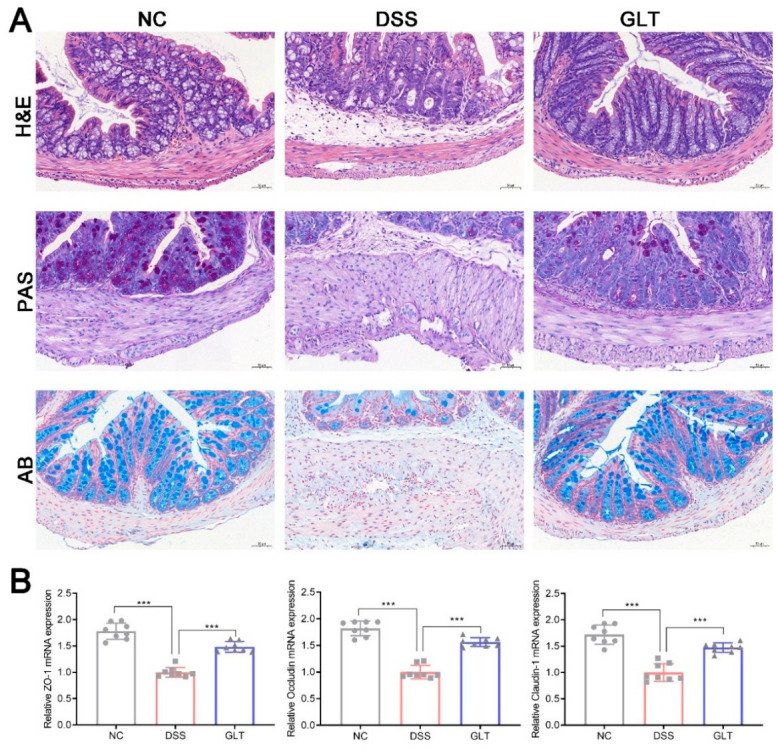
Protective influences of GLT on colonic mucosal architecture and tight junction expression. (**A**) Histological evaluation of mucosal injury (H&E staining) and goblet cell/mucus secretion (PAS and AB staining), Scale bar, 50 μm; (**B**) transcriptional analysis of intestinal barrier-related genes quantified by RT-qPCR, *n* = 8. *** *p* < 0.001 represents significance between each group compared with the DSS group.

The core pathological progression of colitis is characterized by substantial mucosal injury, depletion of goblet cells, muscularis mucosa hyperplasia, and pronounced inflammatory cell infiltration. H&E staining showed that the NC group displayed an intact colonic architecture, featuring well-organized crypts and a distinct glandular structure (Figure 4A and Figure S2). Conversely, the DSS group displayed severe architectural distortion, including crypt loss and extensive inflammatory cell infiltration. GLT intervention markedly attenuated these pathological alterations, preserving crypt integrity and reducing the influx of inflammatory cells. In addition, the result of H&E staining was further confirmed by the results of AB and PAS staining. These findings confirmed that GLT intervention can ameliorate colitis through suppressing the loss of goblet cells and preventing mucus layer injury. These observations were further corroborated by AB and PAS staining results, which specifically highlighted the protective effect of GLT on the intestinal barrier. Compared to the DSS group, GLT-treated colitis mice showed a significant preservation of goblet cells and a reduction in mucus layer degradation. Collectively, these histopathological findings confirm that GLT intervention effectively ameliorates colitis by mitigating mucosal injury and maintaining intestinal secretory function.

In the intestinal epithelium, intercellular connectivity is primarily governed by tight junction proteins, which form the essential architecture for regulating paracellular permeability. To evaluate the effect of GLT intervention on barrier integrity, the mRNA expression levels of colonic Occludin, Claudin-1, and ZO-1 were quantified across the three groups. As shown in Figure 4B, DSS exposure significantly suppressed the transcriptional levels of Occludin, Claudin-1, and ZO-1 compared with the NC group (*p* < 0.001). Conversely, GLT intervention remarkably upregulated the expression of these tight junction proteins in colitis mice (*p* < 0.001). These results indicate that GLT effectively mitigates gut barrier dysfunction via upregulating the transcription of tight junction proteins.

### 3.4. Effects of GLT Intervention on Cecal SCFAs in Colitis Mice

**Figure 5:**
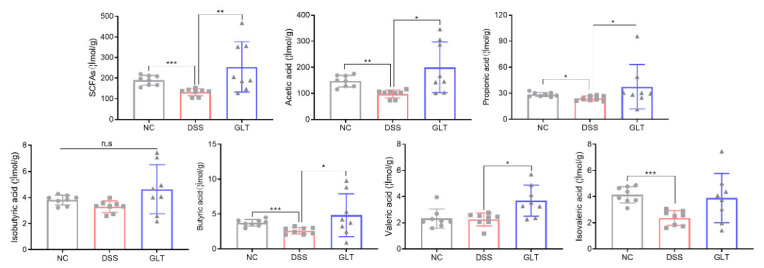
GLT intervention restored cecal SCFA concentrations in mice with DSS-induced colitis, *n* = 8. * *p* < 0.05, ** *p* < 0.01, *** *p* < 0.001 represent significance between each group compared with the DSS group, and n.s represent not significance between each group compared with the DSS group.

SCFAs are primary metabolites derived from the microbial fermentation of indigestible carbohydrates and proteins, playing a pivotal role in maintaining intestinal epithelial homeostasis and improving energy metabolism. To assess the effect of GLT on the gut metabolic profile, cecal SCFA concentrations were quantified (Figure 5). Compared with the NC group, DSS exposure significantly depleted the total SCFA content, specifically reducing the concentrations of acetic, propionic, butyric, and isovaleric acids (*p* < 0.05). In contrast, no obvious alterations were detected in isobutyric and valeric acid levels (*p* > 0.05). Notably, GLT intervention remarkably restored the concentrations of acetic, propionic, and butyric acids in colitis mice (*p* < 0.05). However, GLT treatment did not significantly influence the concentrations of isobutyric and isovaleric acids (*p* > 0.05), suggesting a selective regulatory effect of GLT on primary SCFA production.

### 3.5. GLT Regulated the Gut Microbiota Composition in Colitis Mice

**Figure 6:**
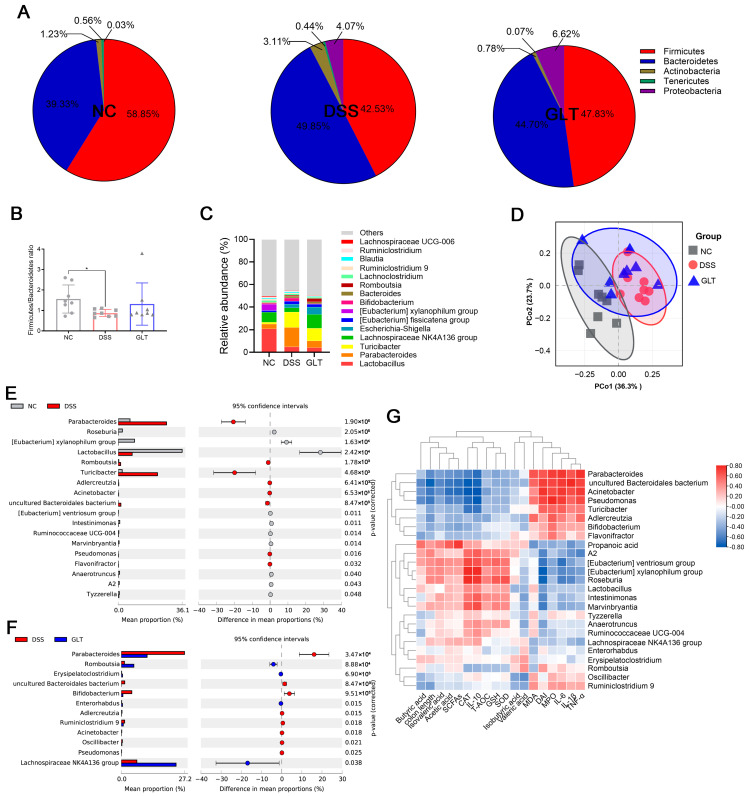
GLT reshaped the gut microbiota composition in colitis mice. (**A**) Relative abundance of gut microbiota at the phylum level, *n* = 8; (**B**) the Firmicutes/Bacteroidota (F/B) ratio, *n* = 8; (**C**) relative abundance of gut microbiota at the genus level, *n* = 8; (**D**) PCoA based on Bray–Curtis distance, *n* = 8; (**E**) differential microbial taxa between the NC and DSS groups, *n* = 8 in each group; (**F**) differential microbial taxa between the DSS and GLT groups, *n* = 8; and (**G**) Spearman’s correlation analysis between differential microbial genera and colitis-associated physiological parameters, *n* = 8. * *p* < 0.05 represents significance between each group compared with the DSS group.

Gut microbiota dysbiosis is widely recognized as a critical factor in the pathogenesis of colitis. Therefore, the gut microbiota composition of the three groups was detected using 16S rRNA sequencing. As shown in Figure S3, the alpha diversity (Chao1 and Shannon indexes) of gut microbiota in the DSS group was significantly lower than that in the NC group (*p* < 0.01). However, GLT intervention significantly elevated the Chao1 and Shannon indexes in colitis mice (*p* < 0.01). At the phylum level, Firmicutes, Bacteroidota, Actinobacteria, Tenericutes and Proteobacteria constituted the dominant microbial taxa, with notable variations in their relative abundances observed among the groups (Figure 6A). Specifically, the Firmicutes/Bacteroidota (F/B) ratio was remarkably diminished in the DSS group compared with the NC group (*p* < 0.05), but GLT intervention showed a trend toward restoring this ratio (Figure 6B). At the genus level, the core microbiota consisted of *Lachnospiraceae_UCG-006*, *Bacteroides*, *Bifidobacterium*, *Blautia*, and *Lactobacillus*, among others (Figure 6C). Furthermore, PCoA based on Bray–Curtis distance revealed a distinct segregation between the NC and DSS groups (Figure 6D). Notably, the GLT group shifted toward the NC group cluster, suggesting that GLT intervention promoted the normalization of the gut microbiota structure and mitigated DSS-caused microbial shifts.

As presented in Figure 6E, DSS challenge induced a profound shift in the gut microbial profile at the genus level. Specifically, the relative abundances of harmful bacteria (including *Parabacteroides*, *Adlercreutzia*, *Acinetobacter* and *Pseudomonas*) were dramatically elevated in the DSS group compared with the NC group. Conversely, beneficial genera (such as *Roseburia*, *Lactobacillus*, and members of the [*Eubacterium*] *xylanophilum group*) were remarkably depleted. Notably, GLT intervention effectively mitigated these dysbiotic changes, significantly reducing the overgrowth of *Parabacteroides*, *Adlercreutzia* and *Acinetobacter* in colitis mice. Furthermore, GLT supplementation selectively enriched several functional taxa, including *Romboutsia*, *Erysipelatoclostridium*, and *Lachnospiraceae NK4A136 group*. These genera were identified as key potential biomarkers following GLT administration (Figure 6F).

To elucidate the potential relationship between gut microbiota composition and colitis-related physiological parameters, a multivariate correlation matrix was constructed based on Pearson correlation coefficients (Figure 6G). The relative abundances of several opportunistic pathogens and pro-inflammatory taxa (including *Parabacteroides*, *Acinetobacter*, *Pseudomonas*, *Turicibacter*, *Adlercreutzia* and *Flavonifractor*) were positively correlated with DAI, pro-inflammatory cytokines (TNF-α, IL-1β, and IL-6) and oxidative stress markers (MPO and MDA). Conversely, these genera exhibited strong negative associations with colon length, SCFAs (acetic, butyric and isovaleric acids), and the antioxidant defense system (including CAT, T-AOC, GSH, and SOD). In contrast, beneficial genera (including *Roseburia*, *Lactobacillus*, *Intestinimonas*, and [*Eubacterium*] *xylanophilum* and [*Eubacterium*] *ventriosum groups*) were positively associated with anti-inflammatory IL-10 levels, antioxidant enzyme activities, and SCFA production, while being inversely related to inflammatory and oxidative indicators. Collectively, these findings suggest that the therapeutic efficacy of GLT against DSS-induced colitis is mechanistically linked to its capacity to rebalance the gut microbiota and modulate associated metabolic and inflammatory profiles.

### 3.6. GLT Modulated Colonic Metabolic Profiles in DSS-Treated Mice

**Figure 7:**
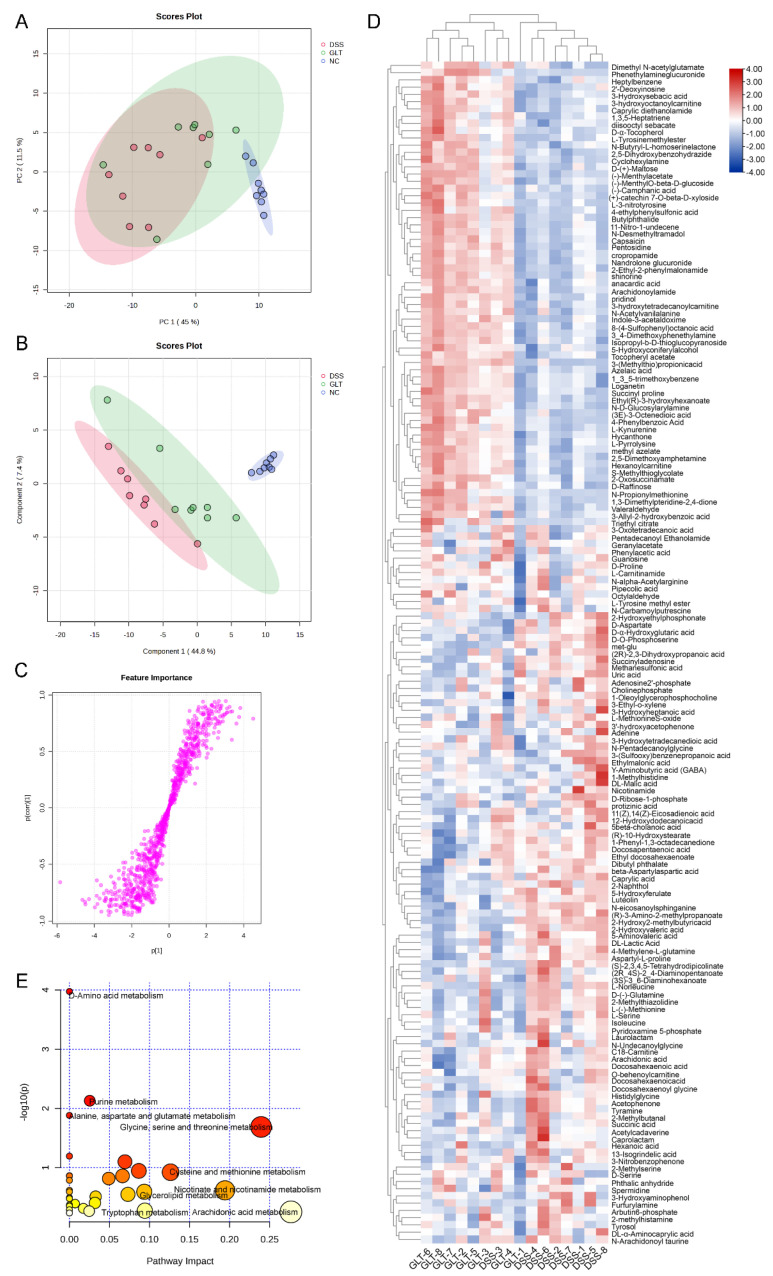
GLT intervention remodeled the colonic metabolic profiles in DSS-induced colitis mice. (**A**) PCA score plot illustrating global metabolic variations, *n* = 8; (**B**) PLS-DA score plot, *n* = 8; (**C**) OPLS-DA S-plot identifying discriminating metabolites between the DSS and GLT groups, *n* = 8; (**D**) heatmap of the relative abundance of differentially expressed metabolites between the DSS and GLT groups, *n* = 8; and (**E**) KEGG pathway enrichment analysis based on the identified discriminating metabolites, *n* = 8.

To further elucidate the metabolic mechanisms by which GLT intervention alleviates colitis-related inflammation and oxidative stress, a non-targeted metabolomics analysis was implemented by UPLC Q Exactive-MS/MS. PCA and PLS-DA were applied to evaluate the global metabolic variations among the three groups. The PCA score plot showed a distinct separation between the NC and DSS groups, suggesting substantial metabolic dysregulation in colitis mice (Figure 7A). Notably, the metabolic profile of the GLT group shifted toward that of the NC group, suggesting that GLT intervention effectively restored colonic metabolic homeostasis. These findings were further corroborated by the PLS-DA model (R2 = 0.927 and Q_2_ = 0.651, Figure 7B), while OPLS-DA S-plots (R_2_Y = 0.829 and Q_2_ = 0.517, indicating that the models had ideal predictability and high interpretability) highlighted the specific discriminating metabolites between the DSS and GLT groups (Figure 7C). A total of 155 differentially expressed metabolites (DEMs) were identified between the DSS and GLT groups, comprising 68 upregulated and 87 downregulated species (Figure 7D). KEGG pathway enrichment analysis revealed that these DEMs were involved in 27 metabolic pathways (Figure 7E). Notably, GLT intervention primarily modulated pathways related to amino acid metabolism (including D-amino acid, alanine, aspartate, glutamate, glycine, serine, threonine, cysteine, and methionine metabolism), as well as lipid and vitamin metabolism (glycerolipid and nicotinate/nicotinamide metabolism) and tryptophan metabolism.

## 4. Discussion

**Figure 8:**
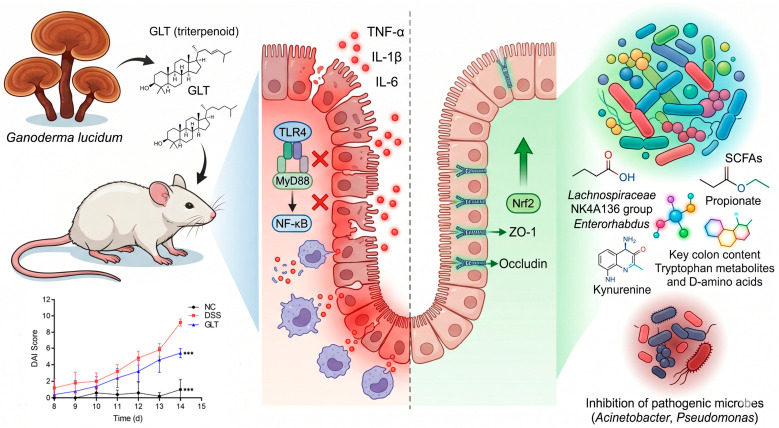
Exposure of GLT attenuates intestinal barrier damage in DSS-treated mice. *** *p* < 0.001 represent significance between each group compared with the DSS group.

Colitis is a chronic inflammatory gastrointestinal disorder that has evolved into a global health challenge due to its escalating incidence and high recurrence rates [10]. Current clinical management primarily relies on anti-inflammatory and immunomodulatory drugs. However, their long-term administration is frequently associated with adverse effects, such as gastrointestinal distress and systemic toxicity, which limit their therapeutic sustainability [11]. Consequently, there is an urgent need for safer alternatives, with natural bioactive compounds and probiotics gaining significant attention for their multi-target health benefits. Among these, triterpenoids are a class of structurally diverse secondary metabolites recognized as the primary bioactive constituents of *G. lucidum*. Although the anti-tumor and anti-inflammatory properties of GLT have been documented [12], the specific mechanisms by which they modulate colitis remain poorly understood. In the present study, we demonstrated that GLT intervention significantly ameliorates DSS-induced colitis (Figure 8). This protective effect is closely associated with the suppression of systemic inflammation and oxidative stress, the elevation of cecal SCFA production, and the comprehensive reshaping of the gut microbiota and colonic metabolic profiles.

Accumulating evidence demonstrates that the DSS-induced colitis model closely recapitulates the clinical manifestations of human colitis, characterized by progressive weight loss, diarrhea, and hematochezia. These parameters serve as gold standards for evaluating the therapeutic potential of natural bioactive compounds. Our results revealed that oral administration of GLT significantly mitigated body weight loss, alleviated diarrhea and fecal occult blood, and lowered DAI scores in colitis mice, underscoring its potent capacity to ameliorate colitis. Mechanistically, DSS-induced chemical damage to the enteric epithelium typically triggers colonic leakage and an exaggerated inflammatory response, facilitating the infiltration of endotoxins and pathogens, thereby further exacerbating organ dysfunction [13]. Therefore, maintaining intestinal homeostasis is fundamentally dependent on tight junction proteins (ZO-1, Occludin, and Claudin-1), which act as a selective physical barrier. Among them, ZO-1, Occludin, and Claudin-1 are the core structural components of intestinal tight junctions in the intestine. A previous report found that the deficiency of ZO-1 exacerbates the damage to the intestinal barrier and prevents the repair of intestinal barrier damage [14]. Occludin is a cell membrane protein that incorporates into the mucosal barrier by organizing into filamentous polymers on the plasma membrane that seal the apical junctional complex of cells [15]. As an integral component of tight junction proteins, Claudin-1 is essential for regulating paracellular permeability and is implicated in the control of cell proliferation and migration [16]. Therefore, elevating the expression of ZO-1, Occludin, and Claudin-1 is the intrinsic pathway to ameliorate colitis. In the present study, GLT intervention remarkably upregulated the expression of these core tight junction proteins, implying that GLT exerts its protective effects by safeguarding the structural integrity of the intestinal barrier and effectively preventing the translocation of inflammatory stimuli.

Exaggerated inflammatory responses are pivotal drivers in the pathogenesis and progression of colitis, typically characterized by an imbalance between pro-inflammatory and anti-inflammatory cytokines [17]. Among these, TNF-α, IL-1β, IL-6, and IL-10 play multifaceted roles in the inflammatory cascades governing intestinal health. Specifically, TNF-α can impair epithelial integrity by promoting myosin light chain kinase expression, thereby increasing intestinal permeability [18]. This process is often amplified by IL-1β derived from activated macrophages, whose aberrant expression exacerbates both local and systemic pathological conditions. Furthermore, IL-6 acts as a central mediator that upregulates pro-inflammatory pathways, including *NF-κB* and *STAT3*, in non-immune cells, making it a critical therapeutic target in colitis management [19]. Conversely, IL-10 serves as a vital anti-inflammatory cytokine—secreted by T cells, dendritic cells, and neutrophils—that maintains immune homeostasis by antagonizing Toll-like receptor (TLR) activation [20]. In the present study, DSS treatment significantly stimulated the secretion of pro-inflammatory cytokines while suppressing IL-10 levels. Notably, GLT intervention effectively reversed these trends, suggesting that GLT exerts its protective effects against DSS-induced colitis by rebalancing the cytokine profile and suppressing excessive inflammatory responses.

In healthy individuals, the gastrointestinal tract harbors a dense and diverse microbial ecosystem that serves as a critical immunological organ. Consistent with prior reports, DSS treatment induced profound gut microbiota dysbiosis, primarily characterized by the depletion of beneficial taxa and the proliferation of opportunistic pathogens. In the present study, the relative abundances of key SCFA-producing genera (including *Lactobacillus*, *Roseburia*, *Ruminococcaceae UCG-004*, *Tyzzerella*, and *Anaerotruncus*) were significantly diminished following DSS exposure [21,22]. Notably, *Lactobacillus* and *Roseburia* are recognized for their capacity to ferment carbohydrates into acetate and butyrate, providing essential energy substrates for colonic epithelial cells and reinforcing tight junction integrity [23,24]. Furthermore, *Anaerotruncus* and *Ruminococcaceae UCG-004* play vital roles in maintaining metabolic homeostasis and alleviating intestinal inflammation through SCFA production [25,26,27]. The observed association between GLT-elevated SCFAs and anti-colitic effects does not prove direct mediation. SCFAs may be one of multiple beneficial mechanisms, and future studies using SCFA depletion (such as antibiotic treatment, germ-free models, or SCFA receptor knockout animals) or supplementation (such as exogenous SCFA administration) are required to confirm causal mediation. Conversely, the DSS group exhibited a marked enrichment of pro-inflammatory and potential pathogenic bacteria, such as *Romboutsia*, *Turicibacter*, *Acinetobacter*, and *Pseudomonas*. *Romboutsia* has been linked to the induction of Th1 and Th17 cell-mediated immune responses, while *Acinetobacter* and *Pseudomonas* possess diverse virulence factors that exacerbate organelle stress and systemic inflammation [28,29,30,31]. Remarkably, GLT intervention effectively reversed these dysbiotic trends by significantly enriching anti-inflammatory and SCFA-producing bacteria. For instance, GLT notably increased the abundance of *Enterorhabdus*, which may modulate bile acid metabolism and immune responses [32,33], and *Lachnospiraceae NK4A136 group*, a potent butyrate producer [34]. In addition, the Firmicutes/Bacteroidota ratio was widely reported to be closely related to glucose and lipid metabolism disorders, but whether its imbalance is related to inflammation has not been confirmed. Although OTU has lower resolution and reproducibility than ASV, these findings suggest that GLT ameliorates colitis by reshaping the gut microbial landscape toward a more homeostatic and fermentative profile.

Integrative analysis of microbiome and metabolomics data provides a comprehensive framework for linking microbial community dynamics with functional metabolic outputs, thereby improving our understanding of host–microbiota interactions and their contribution to health and disease [35,36]. Modulating the colonic metabolic profile represents a critical mechanism by which dietary interventions maintain intestinal homeostasis. Amino acid metabolism, in particular, exerts a profound influence on mucosal integrity, epithelial barrier function, and systemic immunity, all of which are intimately linked to the pathogenesis of colitis [37,38]. While elevated concentrations of certain amino acids, such as arginine and leucine, have been associated with inflammatory exacerbation, the activation of tryptophan metabolism is recognized for its protective effects, primarily through the upregulation of the AhR/IL-22 signaling pathway. While elevated concentrations of certain amino acids (such as arginine and leucine) have been associated with inflammatory exacerbation, the activation of tryptophan metabolism is recognized for its protective effects, primarily through the upregulation of the AhR/IL-22 signaling pathway [39,40]. Specifically, the microbial conversion of tryptophan into metabolites like kynurenine and indole derivatives serves as a key secondary signal for modulating intestinal barrier resilience [41]. In the present study, GLT intervention significantly reshaped colonic metabolic landscapes, notably affecting D-amino acid metabolism (D-aspartate and L-serine); alanine, aspartate, and glutamate metabolism (succinate and 2-oxosuccinamate); and tryptophan metabolism (kynurenine). Furthermore, alterations in beta-alanine metabolism, specifically involving the polyamines spermine and spermidine, were observed. These metabolic shifts likely contribute to the attenuation of inflammatory responses and oxidative stress, thereby fortifying the colonic microenvironment against DSS-induced injury [42]. Although the current study provides robust multi-omics evidence, it predominantly demonstrates associations among microbiota changes, metabolite alterations, and inflammatory outcomes. Because mechanistic validation experiments (such as antibiotic-induced microbiome depletion, fecal microbiota transplantation (FMT), germ-free mouse models, or targeted metabolite supplementation) were not performed, definitive causal relationships regarding the microbiota–metabolite–immune axis cannot be established. Future studies incorporating these functional validations are imperative to elucidate the precise metabolic signaling pathways mediating the efficacy of GLT.

## 5. Conclusions

In the present study, GLT intervention ameliorated DSS-induced colitis in mice, as evidenced by reduced DAI, restored colon length, improved barrier integrity, decreased pro-inflammatory cytokines, enhanced CAT activity, and upregulation of tight junction proteins. 16S rRNA sequencing and metabolomics revealed that GLT reshaped gut microbiota (enriching SCFA-producing genera) and modulated colonic amino acid metabolism. Based on these observed correlations, we speculate that GLT may exert anti-colitic effects partly via gut microbiota and metabolite modulation, as well as inhibition of TLR4/NF-κB and activation of Nrf2 pathways. Direct causality requires future validation using germ-free models or selective inhibitors. Overall, these findings suggest that GLT may have potential as a functional food ingredient for the supportive management of colitis, although further studies in humans are required to confirm its efficacy and safety.

## Data Availability

The original contributions presented in this study are included in the article/Supplementary Materials. Further inquiries can be directed to the corresponding authors.

## Supplementary Materials

The following supporting information can be downloaded at https://www.mdpi.com/article/10.3390/foods15112016/s1, Figure S1: Schematic diagram of this study. Figure S2: Effect of GLT on the alpha diversity of colonic histological score, goblet cells, and mucosal layer thickness in colitis mice. Figure S3: Effect of GLT on the alpha diversity of gut microbiota in colitis mice. Table S1: Primer sequences used for qPCR.
