# Supplementary material for: Integrated Microbiome and Metabolomics Analysis Reveals That Ganoderma lucidum Triterpenoids Ameliorate Colitis Associated with the Modulation of the Gut Microbiota and Metabolic Profiles
Source: Foods. 2026 Jun 4;15(11):2016. doi: 10.3390/foods15112016 (PMC13256906; doi:10.3390/foods15112016)

Supplementary materials

Table S1 Primer sequences used for qPCR.

Gene	Sequence (5’-3’)	Sequence (3′-5′)
ZO-1	GCGGATGGTGCTACAAGTGATG	GCCTTCTGTGTCTGTGTCTTCATAG
Claudin-1	AGGTCTTGCCGCCTTGGTAG	CCAGGACAGGAACAGGAGAGC
Occludin	ATTAACTTCGCCTGTGGATGACTTC	GTTCTCTTTGACCTTCCTGCTCTTC
TLR4	CTGGGGAGGCACATCTTCTG	CCTCTGCTGTTTGCTCAGGA
NF-κB	AAAAACATCCACCTGCACGC	CTGTCATCCGTGCTTCCAGT
Nrf2	CCTCCGCTGCCATCAGTCAGT	TCGGCTGGGACTCGTGTTCA
β-Actin	GATTACTGCTCTGGCTCCTAGC	GACTCATCGTACTCCTGCTTGC

Figure S1 Schematic diagram of this study.

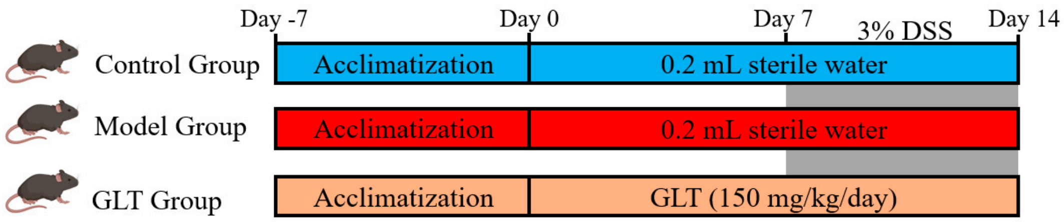

Figure S2 Effect of GLT on the alpha-diversity of colonic histological score, goblet cells, and mucosal layer thickness in colitis mice.

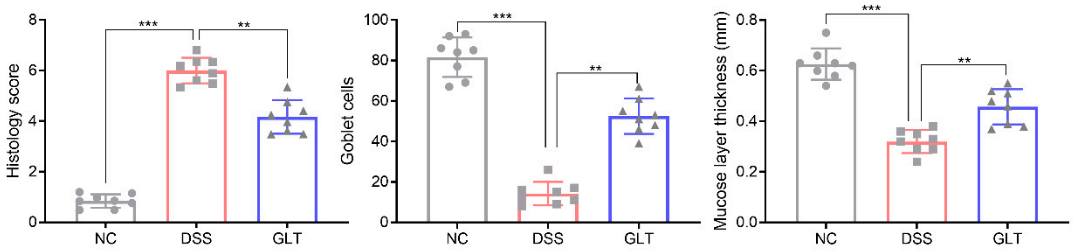

Figure S3 Effect of GLT on the alpha-diversity of gut microbiota in colitis mice

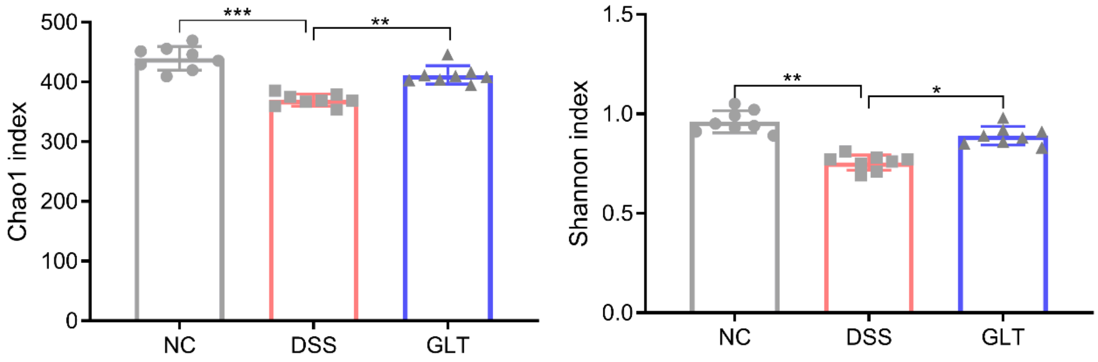

Supplement: Supplementary file 1 [file foods-15-02016-s001.zip › foods-4316369-supplementary.pdf]
